# Hybrid Nanocomposites Based on Poly(3,6-dianiline-2,5-dichloro-1,4-benzoquinone): Synthesis, Structure and Properties

**DOI:** 10.3390/polym16131832

**Published:** 2024-06-27

**Authors:** Svetlana G. Kiseleva, Galina N. Bondarenko, Andrey V. Orlov, Dmitriy G. Muratov, Vladimir V. Kozlov, Andrey A. Vasilev, Galina P. Karpacheva

**Affiliations:** A.V. Topchiev Institute of Petrochemical Synthesis, Russian Academy of Sciences, Leninsky pr., 29, 119991 Moscow, Russia; bond@ips.ac.ru (G.N.B.); avorlov@ips.ac.ru (A.V.O.); muratov@ips.ac.ru (D.G.M.); kozlov@ips.ac.ru (V.V.K.); vasilev@ips.ac.ru (A.A.V.); gpk@ips.ac.ru (G.P.K.)

**Keywords:** oxidative polymerization, dianilinedichlorobenzoquinone, graphene oxide, nanocomposites

## Abstract

Hybrid nanocomposites based on poly(3,6-dianiline-2,5-dichloro-1,4-benzoquinone) (PDACB) in salt form and graphene oxide (GO) have been obtained for the first time, and the significant influence of the preparation method on the composition and structure of nanocomposites and their functional properties has been demonstrated. Nanocomposites were prepared in three ways: via ultrasonic mixing of PDACB and GO; via in situ oxidative polymerization of 3,6-dianiline-2,5-dichloro-1,4-benzoquinone (DACB) in the presence of GO; and by heating a suspension of previously prepared PDACB and GO in DMF with the removal of the solvent. The results of the study of the composition, chemical structure, morphology, thermal stability and electrical properties of nanocomposites obtained via various methods are presented. Nanocomposites obtained by mixing the components in an ultrasonic field demonstrated strong intermolecular interactions between PDACB and GO both due to the formation of hydrogen bonds and π-stacking, as well as through electrostatic interactions. Under oxidative polymerization of DACB in the presence of GO, the latter participated in the oxidative process, being partially reduced. At the same time, a PDACB polymer film was formed on the surface of the GO. Prolonged heating for 4 h at 85 °C of a suspension of PDACB and GO in DMF led to the dedoping of PDACB with the transition of the polymer to the base non-conductive form and the reduction of GO. Regardless of the preparation method, all nanocomposites showed an increase in thermal stability compared to PDACB. All nanocomposites were characterized by a hopping mechanism of conductivity. Direct current (dc) conductivity σ_dc_ values varied within two orders of magnitude depending on the preparation conditions.

## 1. Introduction

The current level of science and technology prompts tasks connected with development and research of new promising materials with a set of required properties. Hybrid nanocomposites, whose constituent elements are conjugated polymers (CP), the unique properties of which are associated with the delocalization of π-electrons along the conjugation chain, as well as carbon nanomaterials are among the most requested materials for cutting-edge technologies. The incorporation of carbon nanomaterials such as graphene oxide (GO) and reduced graphene oxide (RGO) into the structure of nanocomposites has become a common approach in materials science due to the improvement in the properties provided by these carbon components [1,2,3,4,5,6,7,8]. The range of potential practical application of such composites includes the production of supercapacitors [1,3,9,10], batteries [11], membranes [12], sensors and biosensors [13,14], electrochemical current sources [14], fuel cells [15], anti-corrosion coatings [16,17], solar battery [18], etc.

GO is an oxidized derivative of graphene; it has garnered considerable attention [19,20], especially in the field of creating composite materials. The GO surface is a collection of randomly distributed islands of graphene with sp^2^ hybridized carbon bonds, surrounded by fairly large areas of sp^3^ hybridized bonds of oxygen-containing (hydroxyl, epoxy, carboxyl, carbonyl) groups. The latter trigger the layering of GO and make it hydrophilic. This facilitates the dispersion of GO in water, organic solvents, as well as in various polymer matrices, which is an important condition for obtaining composites with improved physical and chemical properties [21]. Due to the presence of a large number of protonogenic oxygen-containing groups on the surface of graphene nanostacks, GO demonstrates good ionic conductivity, which depends on the degree of oxidation of GO and the humidity of the medium and can reach 10^−6^–10^−1^ S⋅cm^−1^ [22,23]. However, unlike graphene, which is characterized by high electronic conductivity, GO is a dielectric, since its production causes the disruption in the continuity of the system of conjugated sp^2^ hybridized carbon bonds of graphene [24].

Being part of hybrid nanomaterials, CPs determine the properties related to electrical conductivity of composites. Moreover, the polymer matrix contributes to the effective stabilization of carbon nanoparticles, thus increasing the overall stability of the nanocomposite. Polyaniline (PANI), its N- and C-derivatives, polythiophene, polypyrrole, etc., are used as CPs in composites [1,2,3,25,26,27,28,29]. The degree of conductivity of this class of polymers depends both on the synthesis conditions and on the type of the dopant, the degree of doping and the size of polyconjugation regions of the forming polymer chain. PANI is most often used as a polymer component due to the simplicity of its preparation, low cost, ease of doping and dedoping processes and its wide range of conductivity [30,31,32]. 

To date, a variety of production methods for nanocomposite materials based on CP and GO has been developed. These include, for example, physically mixing GO with a prepared polymer [33,34,35], grafting macromolecules on the surface of GO [9,36], electrodeposition of a polymer on GO, etc. [10,18,37]. However, the tendency of GO to aggregate makes it difficult to achieve their uniform distribution in nanocomposites, despite a stabilizing polymer matrix. To solve this problem, in situ oxidative polymerization of monomers in the presence of GO is most commonly used. This method is favorable due to its simplicity of instrumentation, the achievement of high dispersion of the carbon filler and scalability [38,39]. The use of ultrasound in the polymerization process ensures a finely dispersed distribution of the inorganic component in the reaction medium and prevents its aggregation [40,41]. During the in situ oxidative polymerization of aniline, the monomer is adsorbed on the GO surface, which plays an important role in the formation of the nanocomposite structure. The binding of aniline to GO can be accompanied by chemical reduction of GO with a partial removal of oxygen-containing groups and the appearance of electronic conductivity [42,43]. At the same time, the preservation of some functional groups prevents the compaction of GO sheets and favors the formation of hydrogen bonds, electrostatic interactions and π-stacking between aromatic structures of RGO and PANI [4,5,43,44,45,46].

Poly(3,6-dianiline-2,5-dichloro-1,4-benzoquinone) (PDACB) is a member of a new family of CPs synthesized by us, the diarylaminodichlorobenzoquinones, which have bulk electroactive side substituents [47]. We studied the kinetics of the polymerization process, the formation mechanism of active polymerization centers and the chemical structure of the formed polymer in neutral form. PDACB-based nanocomposites obtained via oxidative polymerization of the monomer in the presence of GO exhibited high-value and stable electrochemical characteristics in a proton electrolyte [48].

In the present work, hybrid nanocomposites based on PDACB in salt form and GO were prepared for the first time and the significant influence of the preparation conditions of nanocomposites on their structure and functional properties was shown. The results of the study of the composition, chemical structure, morphology, thermal stability and electrical properties of nanocomposites based on PDACB and GO depending on the preparation method are presented. Nanocomposites were prepared in three ways: by ultrasonic mixing of PDACB and GO; via in situ oxidative polymerization of DACB in the presence of GO with partial reduction of GO; and by heating a suspension of previously prepared PDACB and GO in DMF with the removal of the solvent and the formation of PDACB/RGO nanocomposites. Fourier transform infrared (FTIR) and Raman spectroscopy, X-ray phase analysis (XPA), scanning electron microscopy (SEM), thermogravimetric analysis (TGA) and impedancemetry were used to study the resulting hybrid nanocomposites.

## 2. Materials and Methods

### 2.1. Materials

Chloranil (reagent grade) was recrystallized from 1,4-dioxane. Dioxane (reagent grade) was distilled at 101 °C. Aniline (reagent grade) was distilled twice at a residual pressure of 1.33 kPa and a temperature of 50 °C. Ammonium peroxydisulfate (reagent grade) was recrystallized from water at 40 °C. Distilled water was distilled twice. Solutions of HCl (analytical grade) and NH_4_OH (extra pure grade) were used without additional purification.

GO was used in the form of an aqueous suspension. For that, a suspension of graphite oxide in water (10 mg/mL) was dispersed with an ultrasonic dispersant MEF93.T (MELFIZ, Moscow, Russia) for 30 min at 50 °C.

### 2.2. Synthesis of Composites Based on PDACB and GO 

Three different methods were used to obtain composites based on PDACB and GO: ultrasonic mixing of PDACB and GO in an aqueous HCl solution; in situ oxidative polymerization of DACB in the presence of GO; and heating a suspension of previously prepared PDACB and GO in DMF with the removal of the solvent.

#### 2.2.1. Synthesis of PDACB/GO by Mixing PDACB and GO (Method 1)

The PDACB polymer was previously obtained via oxidative polymerization of DACB in an aqueous acid solution in the presence of ammonium peroxydisulfate [47,49]. Weighed portions of 0.1 g of PDACB in salt form and GO 12 wt% of PDACB mass were put into a vessel containing 15 mL of 0.05 M HCl and were sonicated for 25 min while cooling (~5 °C) to achieve uniform dispersion of the components. Then, they were filtered on a Schott filter, and the precipitate was dried in a vacuum dryer. The yield of the final product was 89%.

The resulting composite was labelled as PDACB/GO-1.

#### 2.2.2. Synthesis of PDACB/GO via In Situ Oxidative Polymerization of DACB in the Presence of GO (Method 2)

The monomer, 3,6-dianiline-2,5-dichloro-1,4-benzoquinone (DACB), was obtained via an alkylation reaction of aniline with chloranil following our previously described method [47,49]. The preparation of the reaction mixture for the oxidative polymerization of DACB in the presence of GO was carried out in several stages:

- Two suspensions were prepared separately using ultrasonic dispersion under cooling (~5 °C): 20 mL of GO weight portion (6 (12) wt% of DACB) in water (10 min) and 0.002 mol of DACB monomer in 30 mL of water (20 min).
- A suspension of GO was added to the vessel with the DACB suspension and additionally dispersed for 20 min.
- Then, 10 mL of HCl solution of the calculated concentration were added to the resulting mixture under stirring (to bring the total molarity of the acid in the reaction solution to the required values) and thermostated at 18 °C.

To initiate polymerization, 0.0025 mol of an oxidizing agent—ammonium peroxydisulfate ((NH_4_)_2_S_2_O_8_) (APS)—was added instantaneously to 10 mL of HCl solution. The oxidative polymerization of DACB in the presence of GO continued for 4 h at T = 18 °C and was filtered on a Schott filter. The precipitate was then washed with a 0.2 M HCl solution and dried in a vacuum dryer until constant weight. The yield was 81–85%.

Composites obtained at a GO content of 6 wt% were labelled as PDACB/GO-2.1 and at a GO content of 12 wt% as PDACB/GO-2.2.

#### 2.2.3. Synthesis of PDACB/GO Composites by Heating a Suspension of Previously Prepared PDACB and GO in DMF with the Removal of the Solvent (Method 3)

The PDACB polymer was previously prepared [47,49]. To obtain PDACB/GO composites, a suspension of GO was prepared in 20 mL of DMF containing 0.15 g of PDACB in salt form. The GO content was 6 and 12 wt% relative to the polymer. For homogenization, the suspension was subjected to ultrasonic stirring for 0.5 h at ~5 °C. Then, the suspension was heated to 85 °C and kept at this temperature for 4 h until the solvent was almost completely removed. The resulting powders were dried in a vacuum dryer to constant weight. The yield was 78–82%.

Composites obtained at a GO content of 6 wt% were labelled as PDACB/GO-3.1 and at a GO content of 12 wt% as PDACB/GO-3.2.

**Scheme 1:**
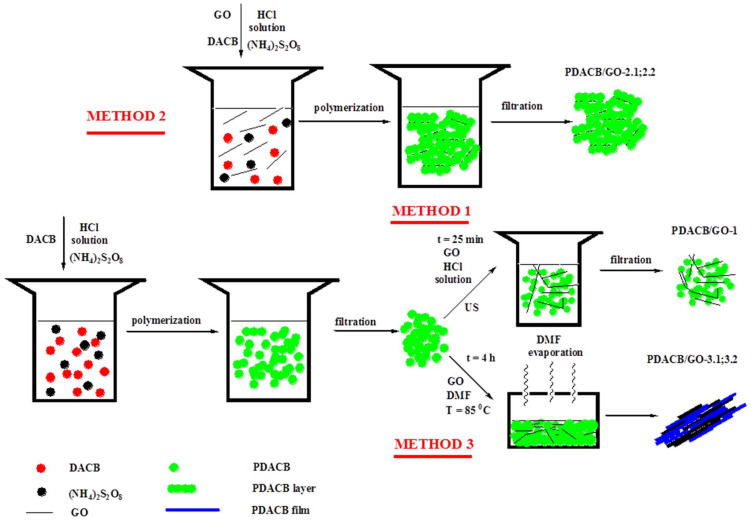
Methods of the preparation of nanocomposites based on PDACB and GO.

Three methods for the preparation of composites based on PDACB and a GO are shown in Scheme 1.

### 2.3. Materials Characterization

Fourier transform infrared (FTIR) spectra were recorded in the ATR mode using a HYPERION-2000 IR microscope (Bruker, Karlsruhe, Germany) coupled with a Bruker IFS 66 V/s FTIR spectrometer (Karlsruhe, Germany) (Ge crystal, scan 100, resolution 2 cm^−1^, range 600–4000 cm^−1^). Optical density was D = lgI_0_/I.

Powder X-ray diffraction (XRD) analysis was performed using a diffractometer “Difray” 401 (Scientific Instruments Joint Stock Company, Saint-Petersburg, Russia) with Bragg–Brentano focusing, using Cr-Kα (wavelength: 0.22909 nm) radiations

Thermogravimetric analysis (TGA) was carried out in the range of 30–450 °C on a Discovery TG TM unit (TA Instruments, New Castle, DE, USA) with a heating rate of 15 °C/min and an N_2_ flow rate of 10 mL/min. The sample weight was 2–4 mg.

FE-SEM images were taken using a Zeiss Supra 25 FE-SEM field emission scanning electron microscope (Carl Zeiss AG, Jena, Germany). The image resolution was 1–2 nm.

Raman spectra were recorded on a Senterra II Raman spectrometer (Bruker, Karlsruhe, Germany) using a laser with a wavelength of 532 nm and a power of 0.25 mW, as well as a spectral resolution of 4 cm^−1^.

Kinetic studies of the oxidative polymerization of DACB were carried out by the potentiometric method using a 4-channel ion meter “Expert-001–3(0.4)” (Russia) with the accuracy of EMF = ±1.5 mV, recording changes in EMF in correlation with the reaction time. A redoxmetric electrode ERP-105 (Moscow, Russia) was used as an electrode. As part of the kinetic studies, the polymerization conditions had the following parameters: [DACB] = 0.03 mol/L; [APS]/[DACB] = 1.25; [HCl] = 0.5 mol/L; T = 18 °C. The amount of GO was calculated in weight percentage of DACB.

The ac conductivity was measured with 6367A precision LCR meter (Microtest Co., New Taipei City, Taiwan) in the frequency range of 0.25 Hz–1.0 MHz.

## 3. Results and Discussion

### 3.1. Structure of PDACB/GO Composites

**Scheme 2:**
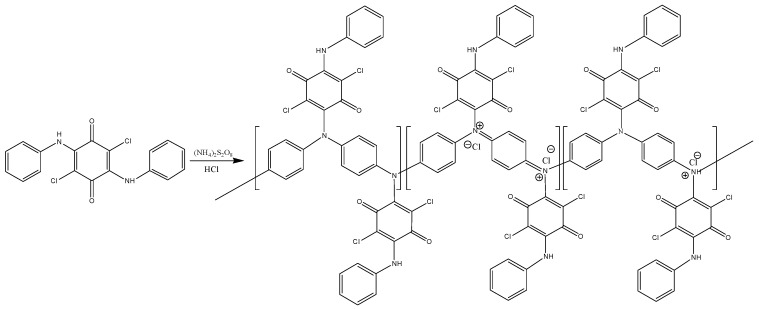
The structure of PDACB as cast in salt form.

**Figure 1:**
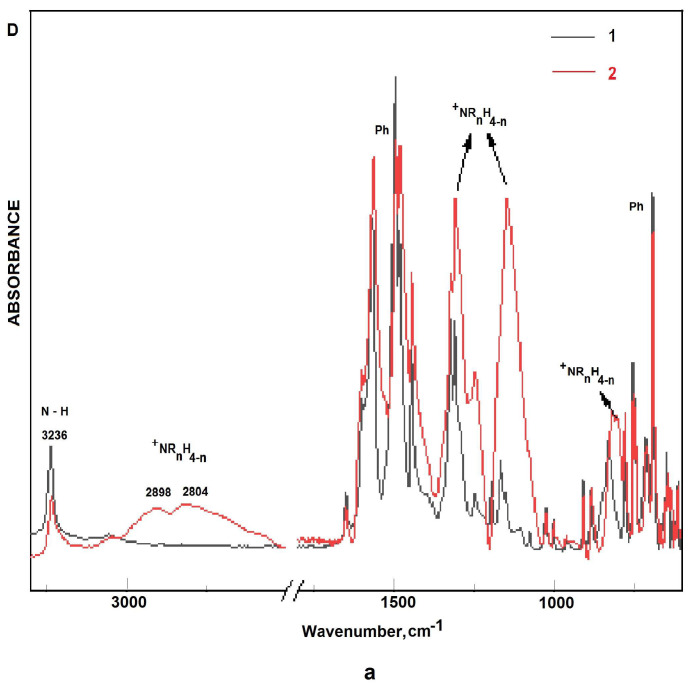

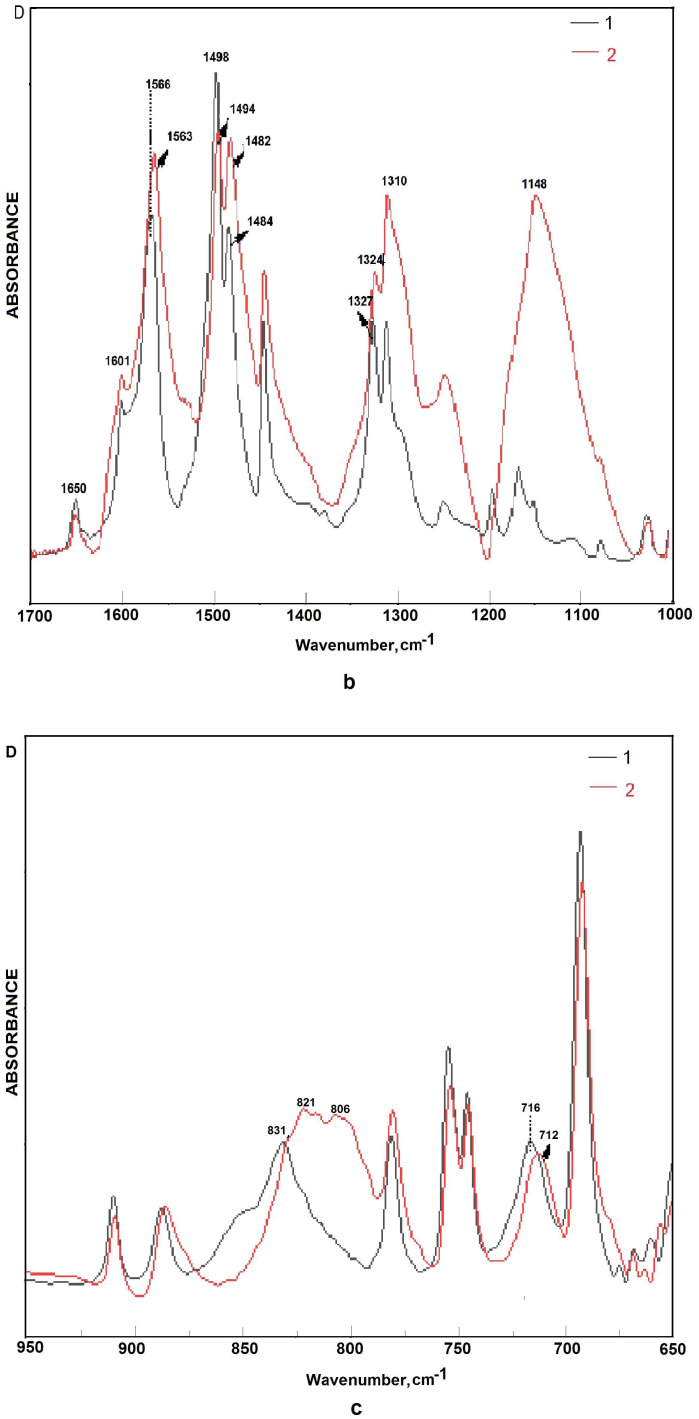
(**a**)—FTIR spectra of PDACB in a neutral (1) and salt (2) forms; (**b**)—detailing of the spectrum in the region of absorption bands of stretching vibrations of C = C(Ph), N-C bonds; (**c**)—detailing in the region of absorption bands of δCCH bending vibrations in aromatic rings.

Three methods were used to obtain nanocomposites: ultrasonic mixing of PDACB and GO; in situ oxidative polymerization of DACB in the presence of GO with partial reduction of GO; and heating the suspension of previously prepared PDACB and GO in DMF with the removal of the solvent and the formation of PDACB/RGO nanocomposites. In this work, the PDACB polymer in a conductive salt form was used as a polymer component. In our previously published works, we established the chemical structure of PADCB in its base neutral form [47,49]. Therefore, structural features of PDACB in salt form (Scheme 2) were studied in the first place. Figure 1a–c shows FTIR spectra of PDACB in neutral and salt forms. Bands characterizing aromatic and quinone rings, N-H and N-C bonds were present in both spectra. The only difference in the spectra was that the bands of ammonium cations at 1310 and 1148 cm^−1^ were very intense in the spectrum of the salt form of the polymer. Broad, diffuse bands in the region of 2900–2800 cm^−1^ indicate that ammonium cations were interconnected by hydrogen bonds.

**Scheme 3:**
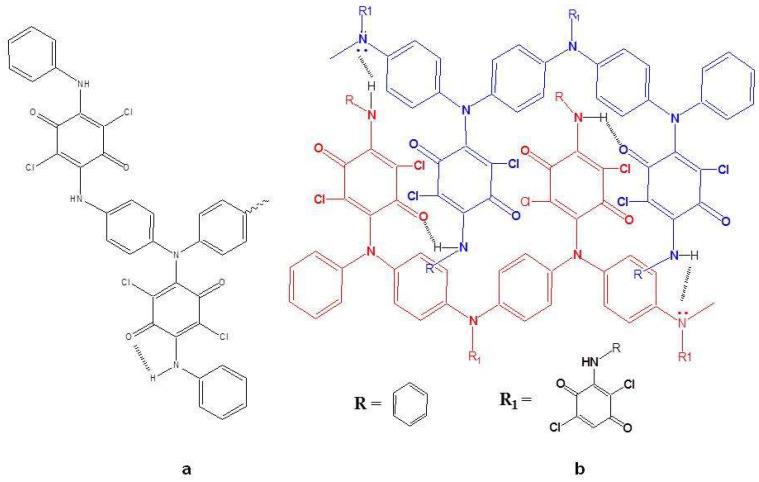
Hydrogen bonds in the structure of PDACB: (**a**) intramolecular; (**b**) interchain.

As can be seen in Figure 1b, bands associated with stretching vibrations of C=C bonds in aromatic rings (1600–1480 cm^−1^) and N-C bonds (1327 cm^−1^) in the spectra of the salt form of the polymer were shifted by 1–3 cm^−1^ towards longer waves, and their intensity was increased. The only exception was the 1494 cm^−1^ band in the spectrum of the salt form of the polymer, the intensity of which decreased. This band was the short-wave component of the doublet (1482–1494 cm^−1^) characterizing the symmetric valence vibrations (ν_s_ C=C(Ph)), which were strongly influenced by the nitrogen atoms in the 1,4-substitution. The difference in the behavior of these bands was due to the different distance of the C=C bonds of the aromatic ring from the substituent. Bands associated with stretching vibrations of the N-H bond in the amino (not ammonium) form (3236 cm^−1^), bands of C=O bonds in the quinone ring (1650 cm^−1^), as well as bands associated with bending vibrations of the quinone ring at 909 and 712 cm^−1^ [47,49] were, on the contrary, reduced in intensity and broadened. Both of these factors indicate the presence of salt form of two types of N-H bonds—free and associated. This leads to different types of conformation of the main chain, i.e., 1,4-N-substituted aromatic rings of the main chain, which must be turned at different angles in units with and without hydrogen bonds [50,51]. The schematic representation of possible intramolecular and intermolecular hydrogen bonds is shown in Scheme 3.

Figure 1c confirms that the band at 831 cm^−1^ in the spectrum of the neutral polymer corresponding to out-of-plane bending vibrations of δCCH in 1,4-substituted aromatic rings in the spectrum of salt form shifted to longer wavelengths, became broader and splits, producing two peaks at 821 cm^−1^ and 806 cm^−1^ [52]. At the same time, the peaks at 693 and 750 cm^−1^ corresponding to δCCH bonds inside monosubstituted aromatic rings showed minor changes in salt form, namely weak shifts and a decrease in their intensity. This may have been due to the degree of rotation of monosubstituted aromatic rings in the side chain around the N–C bond, which was higher than that in 1,4-N-substituted aromatic rings of the main chain, resulting in more uniform conformations in side groups compared to the main chain. Thus, the presence of associated ammonium cations in the structure of salt form of PDACB leads to a more heterogeneous conformational set of the main chain [47,49].

**Figure 2:**
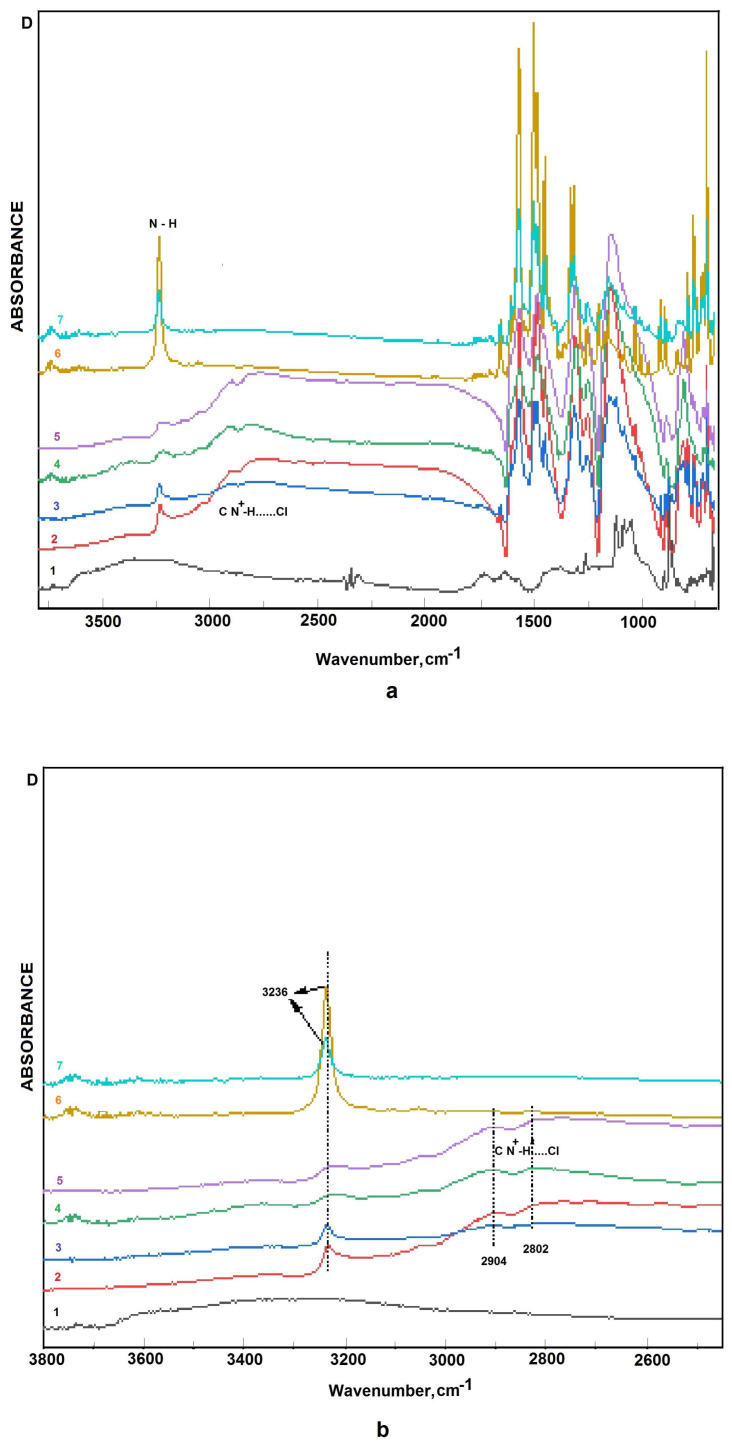

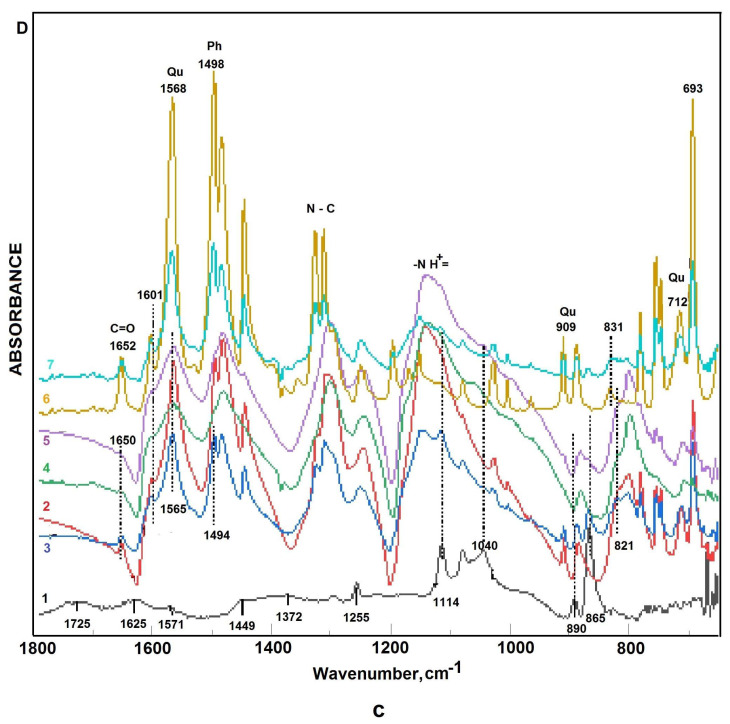
(**a**)—FTIR spectra of PDACB, GO and PDACB/GO composites: GO (1); PDACB (2); PDACB/GO-1 (3); PDACB/GO-2.1 (4); PDACB/GO-2.2 (5); PDACB/GO-3.1 (6); PDACB/GO-3.2 (7); (**b**)—spectrum detailing in the region of 3800–2500 cm^−1^; (**c**)—spectrum detailing in the region of 1800–650 cm^−1^.

Figure 2a–c and 4 help evaluate the structural changes that occurred in the salt form of PDACB during the formation of composites with GO, depending on the synthesis conditions. Figure 2 shows the FTIR spectra of PDACB/GO composites obtained by various methods.

The IR spectrum of GO in Figure 2a demonstrates the structure of a typical carbon material with signs of significant oxidation. The broad band with a peak at around 3300 cm^−1^ was attributed to stretching vibrations of H-O-H bonds in associated water molecules on the surface of GO carbon particles. Weak broad bands at 1625 and 1372 cm^−1^ corresponding to νC-C and COO in GO, respectively, confirm the graphene-like structure of GO, which is a system of condensed aromatic rings with oxidized terminal carbons [53,54]. These bands, in accordance with the selection rules of the theory of vibrational spectroscopy in IR spectra, had low intensity and were analogues of very intense *G* and *D* lines in Raman spectra, respectively, corresponding to sp^2^ and sp^3^ hybridized carbon atoms. The remaining bands in the IR spectrum of GO belonged to C=O (1725 cm^−1^) and C-O (1255, 1114, 1065, 1043, 890, 865 cm^−1^) bonds, with the most intense bands at 1114 and 865 cm^−1^ characterizing C-O epoxy bonds in strained three-membered rings. Therefore, it is possible to postulate the presence of oxidized groups in GO, including carboxyl and epoxy groups [54,55,56].

**Figure 3:**
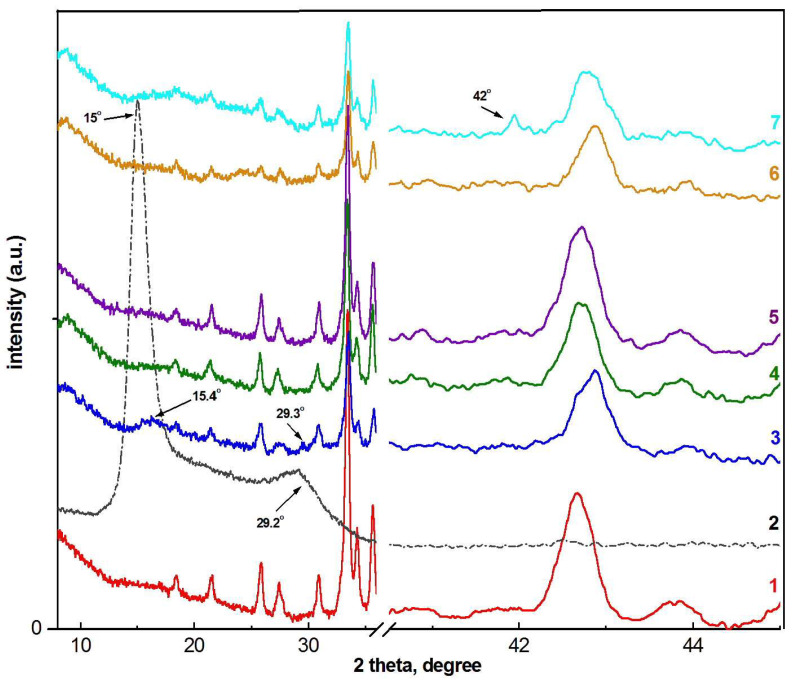
Diffraction patterns of PDACB, GO and PDACB/GO composites: PDACB (1); GO (2); PDACB/GO-1 (3); PDACB/GO-2.1 (4); PDACB/GO-2.2 (5); PDACB/GO-3.1 (6); PDACB/GO-3.2 (7).

The IR spectrum of the PDACB/GO-1 composite, obtained by mixing the polymer and GO (Figure 2, spectrum 3), contained bands of both PDACB and GO; however, it was not an additive overlap of the polymer and GO spectra according to the composition. This was indicated by shifts of the maxima of the PDACB bands towards longer wavelengths and significant changes in the relative intensities of the bands in the absorption region of stretching vibrations of N-C bonds (1300 cm^−1^) and the stretching vibrations of the C=C bonds in the aromatic rings of PDACB (1500 cm^−1^) [57,58]. Oxygen-containing groups in the composition of GO also appeared in the spectrum of PDACB/GO-1 (Figure 2, spectrum 3), and the maxima of their absorption bands were slightly shifted towards shorter wavelengths compared to the spectrum of GO. Such behavior of bands of aromatic rings in PDACB and oxidized groups of GO in the spectrum of the composite can only be explained by the presence of a significant interaction between aromatic rings of the polymer and GO [44,59], and it is the terminal oxidized aromatic rings of GO that take the greatest part in this interaction [60,61]. A decrease in the intensity of the IR spectrum in the region of hydrogen bond vibrations 1700–2800 cm^−1^ also indicates the predominant role of π-π stacking [43]. The interaction of GO layers with the polymer was also proven both by a significant decrease in the height of the reflection peaks of PDACB and GO and their shifts in the diffraction pattern of the sample obtained by mixing the components (Figure 3 diffraction pattern 3) [57,59,62,63].

**Scheme 4:**
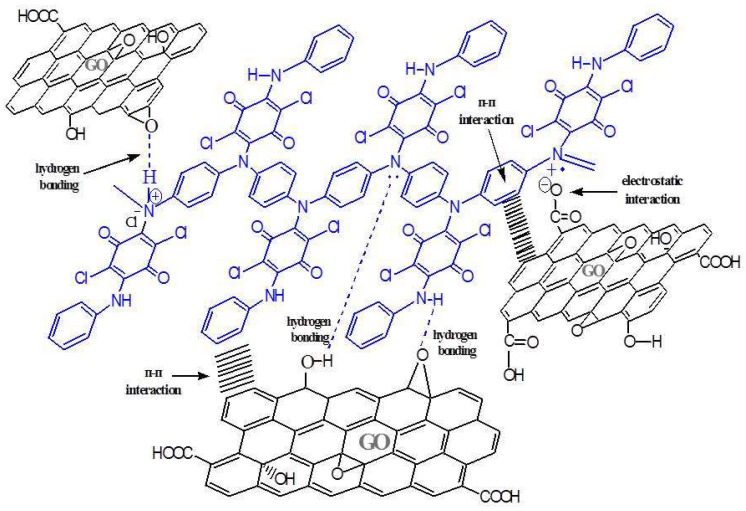
Types of interaction between the polymer and GO.

In this case, there was no reduction of GO, as evidenced by the preservation of absorption bands at 1114 and 865 cm^−1^, which characterized the C-O bonds of epoxy groups in the IR spectra of PDACB/GO-1. This was also confirmed by the presence of a broad reflection peak in the diffraction pattern of the composite in the region of 2θ = 15.4° from the plane (001) of GO and a shoulder in the region of 2θ = 29.3°. The low intensity of the peaks was due to the low content of GO in the composite and the strong interaction between its components [3,57]. The types of interactions between the polymer and GO are schematically presented in Scheme 4.

**Figure 4:**
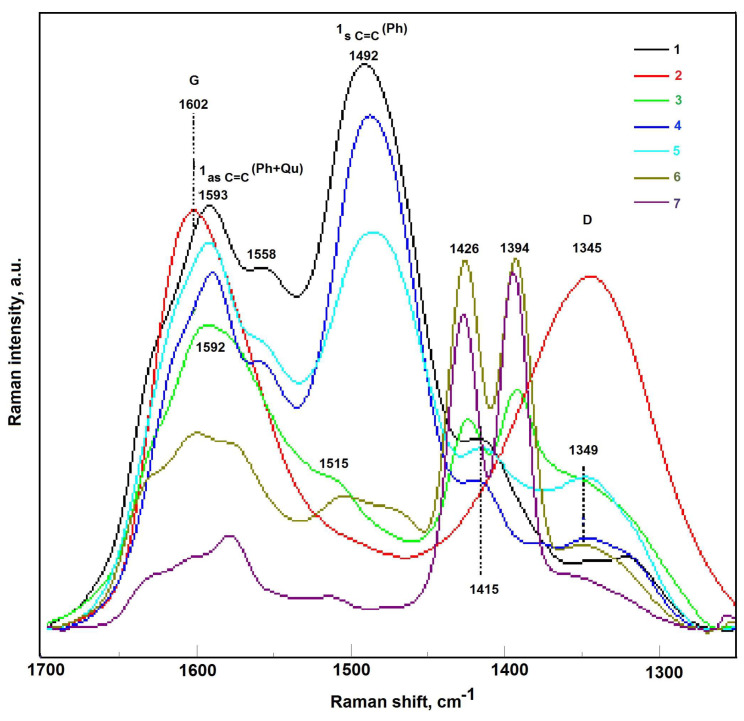
Raman spectra of PDACB, GO and PDACB/GO composites: PDACB (1); OG (2); PDACB/GO-1 (3); PDACB/GO-2.1 (4); PDACB/GO-2.2 (5); PDACB/GO-3.1 (6); PDACB/GO-3.2 (7).

The conclusion about the interaction of the components of the PDACB/GO-1 composite is based on the analysis not only of the IR spectra (Figure 2), but also the Raman spectra (Figure 4), where, according to the selection rules, polar bonds with a large dipole moment (C-O and C=O) do not appear. In this case, -C=C-C=C bonds with small dipole moments were easily polarized and produced very intense signals.

The Raman spectrum of PDACB in salt form (Figure 4, spectrum 1) showed two strong signals at 1593 and 1492 cm^−1^ from the asymmetric and symmetric vibrations of C=C bonds in the aromatic rings of the polymer, respectively. The first one (at 1593 cm^−1^) was significantly contributed to by the C=C bonds in the quinone rings, whereas the second one (at 1492 cm^−1^) corresponded exclusively to vibrational modes in the phenyl rings. Each of these signals had a well-defined long-wavelength shoulder: 1558 cm^−1^ for the former and 1415 cm^−1^ for the latter. This reflects the presence of two types of aromatic rings in the samples: rings with vibrational modes of 1593 and 1492 cm^−1^ have a higher π-electron density, whereas rings characterized by signals at 1558 and 1415 cm^−1^ have a lower π-electron density due to delocalization through the electron pair of nitrogen atoms with the HCl dopant present in salt form of the polymer. Such non-covalent binding leads to the appearance of chain sections with an uneven distribution of electrons in the conjugation system and, consequently, the appearance of new signals from C=C bonds in Raman spectra [43].

The Raman spectrum of GO (Figure 4, spectrum 2) is represented by two signals, *G* (1602 cm^−1^) and *D* (1345 cm^−1^), which are typical for Raman spectra of any carbon material and characterize sp^2^ and sp^3^ carbon atoms, respectively. The similar integrated intensity of both signals proves that the number of sp^2^ carbon atoms forming proper aromatic or quinoid cycles inside a carbon particle is close to the number of sp^3^ carbon atoms in oxidized terminal rings [64].

**Scheme 5:**
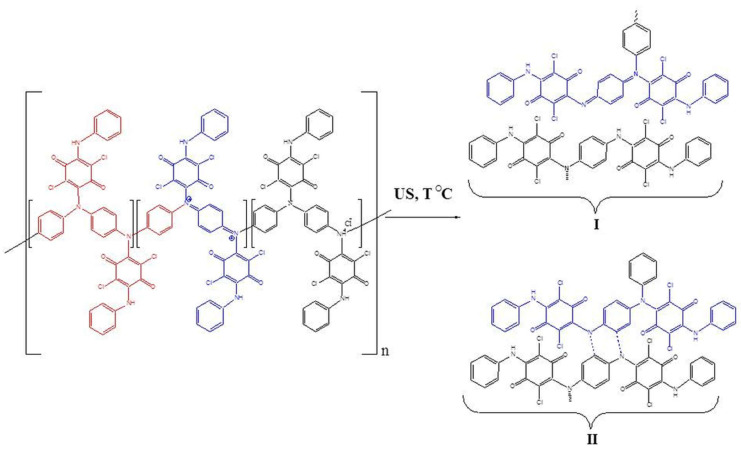
Scheme of formation of low-molecular-weight (I) and cross-linked (II) fragments.

The Raman spectrum of the PDACB/GO-1 composite (Figure 4, spectrum 3) showed a significantly broadened and weakly split band at 1592 cm^−1^, which included the signal from the νC=C phenyl and quinone rings of the polymer and the *G* signal of GO from sp^2^ carbon atoms. The *D* signal (1345 cm^−1^) from the sp^3^ carbon atoms of GO appeared in the form of a weak broad shoulder. The most intense signal from stretching symmetric vibrations of aromatic rings in the spectrum of PDACB (1492 cm^−1^, spectrum 1) was absent in the spectrum of PDACB/GO-1 (spectrum 3). Only a weak shoulder appeared at 1515 cm^−1^. What attracts the most interest here is the appearance of two new lines at 1426 and 1394 cm^−1^ of average intensity, located in the intermediate region between the signals from the sp^2^ and sp^3^ carbon atoms. This could have been due to the appearance of strong interactions between oxidized aromatic rings of GO and aromatic rings of the polymer with the participation of electron pairs on oxygen atoms from GO and nitrogen atoms from PDACB [64]. These signals may have also resulted from the formation of cross-linked fragments of PDACB, because ultrasonication of an aqueous suspension of PDACB and GO during the preparation of PDACB/GO-1 is accompanied by processes of cavitation and local overheating [65]. This can induce both a decrease in the degree of PDACB doping and the breaking of individual bonds followed by the formation of low-molecular-weight “fragments” (I) and “phenazine-like” structures cross-linked as a result of interchain interaction (II) [66,67], as shown in Scheme 5.

Thus, composites obtained by mixing components were characterized by strong interactions between PDACB and GO due to both the electrostatic interaction of oppositely charged fragments and the formation of hydrogen bonds and π-stacking.

The IR spectra of the PDACB/GO-2.1 and PDACB/GO-2.2 composites (Figure 2, spectra 4 and 5) demonstrated all main absorption bands of PDACB. However, the bands that characterize N-H bonds (3232 cm^−1^), N-C (1300 cm^−1^), as well as C=C bonds in aromatic rings (1600–1450 cm^−1^), became broader and shifted to longer wavelengths. This may be have been to a stronger influence of ammonium cations on the structure of conjugated phenyl and quinoid rings of the polymer due to hydrogen bonding, electrostatic forces and π-stacking (Scheme 3). A sharp decrease in intensity, splitting and shifts to short wavelengths of the bands at 692 and 750 cm^−1^ associated with δCCH bonds inside monosubstituted aromatic rings indicates conformational changes in the side polymer chain. This is was a consequence of the interaction of PDACB and GO, where the latter could, in this case, act as a template for growing macromolecules with the formation of a polymer layer [68].

**Figure 5:**
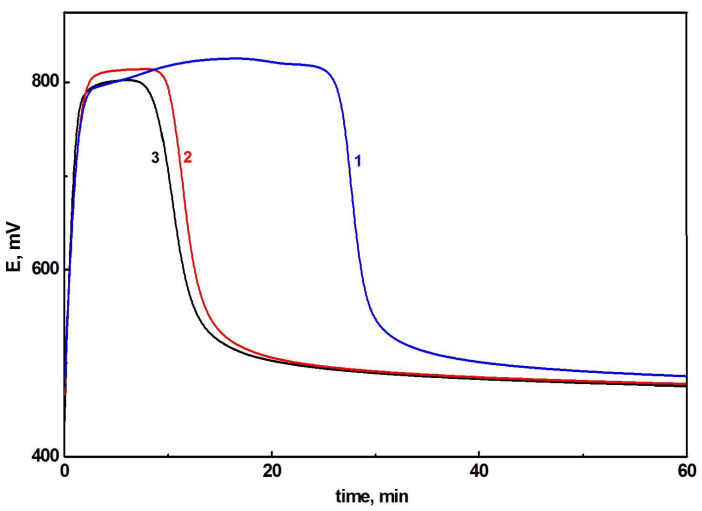
Changes in redox potential over time during oxidative polymerization of DACB: in the absence of GO (1); in the presence of 6% GO (2) and 12% GO (3).

The most intense band associated with the C-O bonds (865 cm^−1^) of the epoxy group in the GO disappeared in the spectrum of PDACB/GO-2.1 (Figure 2, spectrum 4). This can be explained by the fact that GO, having a low reduction potential (−0.94 V vs. NHE), can take part in the process of oxidative polymerization of DACB, being reduced in the process [43,69,70,71]. This was confirmed by kinetic studies of the polymerization process of DACB in the presence of GO (Figure 5). There was an increase in the rate of polymerization in the presence of GO and a shortening of the induction period by almost 2.5 times.

**Scheme 6:**
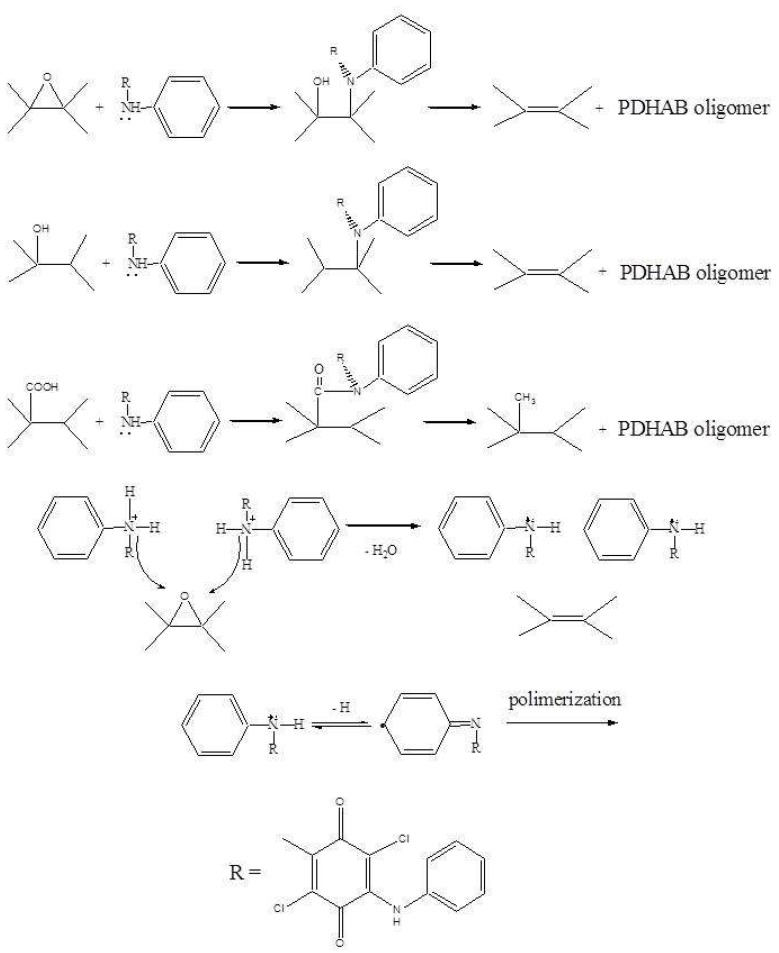
Options of interaction of the monomer with the GO surface.

Possible options of the interaction of the monomer with the GO surface accompanied by chemical reduction of GO are given in Scheme 6.

**Scheme 7:**
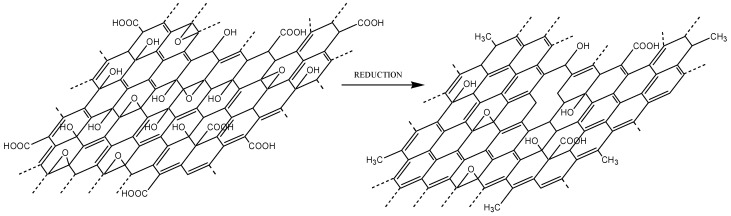
Reduction of GO.

As a result, the GO structure underwent significant changes, as evidenced by the IR spectra of PDACB/GO-2.1 showing the disappearance of absorption that characterized C-O bonds of epoxy groups on the surface of GO (865, 1114 cm^−1^) (or only a trace amount remained) (Scheme 7) [54,55,56,72].

The resulting oligomers, being adsorbed on the GO surface, polymerized in acidic media in the presence of ammonium persulfate according to “head to tail” addition method [43,61]. 

However, a very weak band corresponding to C-O epoxy groups appeared in the IR spectrum of PDACB/GO-2.2 (Figure 2, spectrum 5), which means that not all oxygen-containing groups of GO underwent chemical reduction. This is also indicated by a slight increase in the intensity of the spectrum in the region of 1700–2800 cm^−1^, typical of hydrogen bonds, whose formation is contributed by oxygen-containing groups of GO. Moreover, a higher intensity of the “electronic-like” band in the region of 1148 cm^−1^ (δ CH; ν-NH^+^ =) shifted towards lower wavenumbers, indicates a higher degree of PDACB/GO-2.2 doping and additionally contributed to by residual terminal carboxyl groups of GO that have not undergone chemical reduction [57].

The Raman spectra of the PDACB/GO-2.1 and PDACB/GO-2.2 composites (Figure 4, spectra 4 and 5) were practically no different from the PDACB spectrum. The presence of GO in them was indicated by the *D* line, which had significantly lost intensity and was shifted to the short-wavelength region of the spectrum by 4 cm^−1^ (1349 cm^−1^). Line *G* coincided with the vibrational mode ν_as_ C=C(Ph+Qu) in the polymer spectrum (1593 cm^−1^). In other words, in this case, polymer chains formed on the interphase surface “envelop” GO, preventing its structural features from being recorded in the Raman spectrum. A significant decrease in the intensity of the signal at 1593 cm^−1^ indicates the interaction of PDACB with reduced GO due to π-stacking. This is also indicated by a decrease in the intensity of reflection peaks in the diffraction patterns of the composites (Figure 3, diffraction patterns 4 and 5) compared to PDACB (Figure 3, diffraction pattern 1) [3,62]. The absence of GO reflection peaks indicates the partial reduction of GO.

Therefore, in the process of preparing the PDACB/GO-2.1 and PDACB/GO-2.2 composites via in situ oxidative polymerization of DACB fixed on the GO, the latter participated in the redox process, and a polymer layer was formed on its surface [68,70,73].

**Figure 6:**
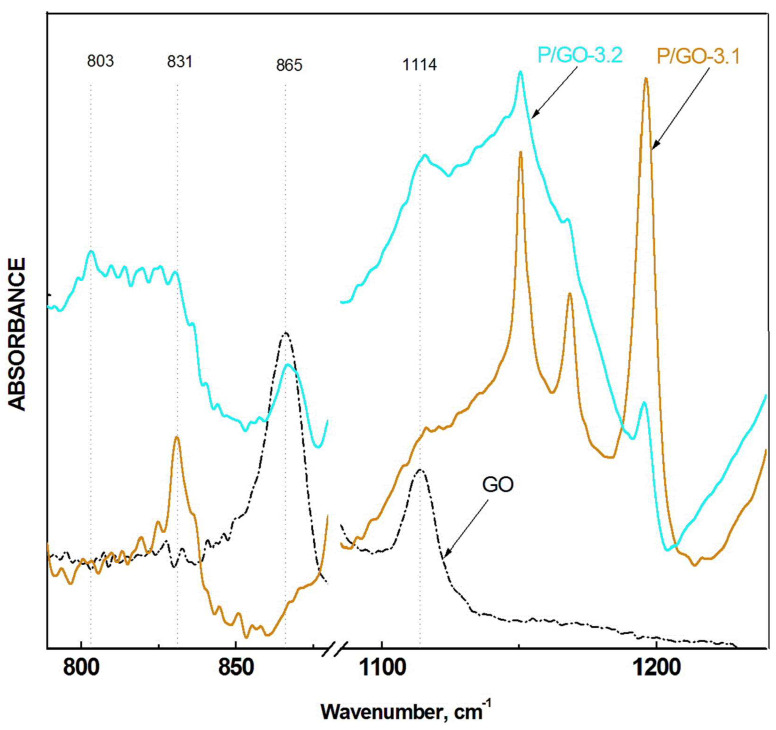
IR spectra of GO (dash-dotted), PDACB/GO-3.1 (orange), PDACB/GO-3.2 (cyan).

The synthesis of PDACB/GO-3.1 and 3.2 composites based on the prepared polymer and GO implies the post-reduction of GO → RGO. The IR spectra of these samples (Figure 2, spectra 6 and 7) were considerably different from the spectra of samples obtained via in situ polymerization in the presence of GO (Figure 2, spectra 4 and 5). Under prolonged heating of the suspension of PDACB and GO in DMF, alongside the reduction of GO, when the solvent evaporates, “dedoping” of PDACB (removal of the HCl dopant) also occurred. The IR spectra (Figure 2c, spectra 6 and 7) confirm this, demonstrating the absence of an absorption band in the region of 800 cm^−1^, which characterizes ammonium cations, and a shift of the band at 821 cm^−1^ associated with out-of-plane bending vibrations δCCH of 1,4-substituted aromatic rings to 831 cm^−1^, as observed in the spectrum of PDACB in neutral form (Figure 1a). The IR spectrum of PDACB/GO-3.1 (Figure 2, spectrum 6) showed the disappearance of spectral features of GO, which indicates the reduction of GO. There iswas however, no complete reduction of GO. This is evidenced by the absence of reflection peaks corresponding to RGO planes in diffractogram 6 in Figure 3. The formation of RGO was observed in the sample labelled as PDACB/GO-3.2. Diffractogram 7 in Figure 3 shows a distinct reflection peak corresponding to the 100 RGO plane [74,75,76,77]. Its low intensity is associated with the low content of GO in the sample volume and strong interaction with the polymer [3,57]. At the same time, the IR spectrum of PDACB/GO-3.2 (Figure 2a, spectrum 7) contained weak bands corresponding to the epoxy groups of GO (865 and 1114 cm^−1^) (Figure 6), indicating their incomplete reduction. This suggests the presence of residual amount of unreduced GO, which, acting as a dopant, ensures doping of the polymer layer despite the removal of HCl [57]. In the IR spectrum of the PDACB/GO-3.2, this was shown by the presence of a weak band at 803 cm^−1^ as well as broadening and splitting of the band in the region of 1070–1200 cm^−1^ (δ CH; ν-NH^+^ =) (Figure 6).

The presence of a residual amount of unreduced GO in the PDACB/GO-3.2 composites was confirmed by the presence of the composite of a very broad weak reflection peak in the diffraction pattern in the region of 2θ = 15.6–25° (Figure 3, diffractogram 7) which was a superposition of reflection peaks from the (001) plane of GO [17,74] and (002) planes of RGO [61,75,76,77].

Thus, based on the obtained results, a conclusion can be made that when nanocomposites are synthesized by the solvothermal method in the presence of 12% GO, a mixture PDACB/GO and PDACB/RGO nanocomposites is formed, where PDACB is present in its base non-conductive form. A general decrease in the intensity of diffraction peaks confirms the formation of a hybrid material and the interaction between its components [27,59,77].

In the Raman spectra of PDACB/GO-3.1 and 3.2 composites (Figure 4, spectra 6 and 7), there were two intense signals at 1426 and 1394 cm^−1^, which were absent in the PDACB/GO-2.1 and PDACB/GO-2.2 spectra (Figure 4, spectra 4 and 5). This, as in the case of PDACB/GO-1 (Figure 4, spectrum 3), may be a result of the formation of cross-linked “phenazine-like” fragments of PDACB (Scheme 4) under ultrasonication during sample preparation by the solvothermal method. Moreover, there was a sharp drop in the intensity of signals from aromatic and quinoid rings of PDACB (1593 and 1492 cm^−1^), and the recorded low-intensity signals were split and blurred, being a superposition of bands corresponding to GO and PDACB in the composite. This may be due to the formation of thin films containing a dispersion of carbon material in a polymer matrix during sample preparation.

According to FTIR, Raman and XRD data, it can be concluded that during the synthesis of PDACB/GO-3.1 and 3.2, the GO → RGO transformation occurred as a result of the reduction of terminal oxidized groups (especially epoxy groups) with the formation of unsaturated C=C bonds. At the same time, fairly strong binding took place between the polymer and GO, accompanied by the emergence of new types of conjugated double bonds. 

**Figure 7:**
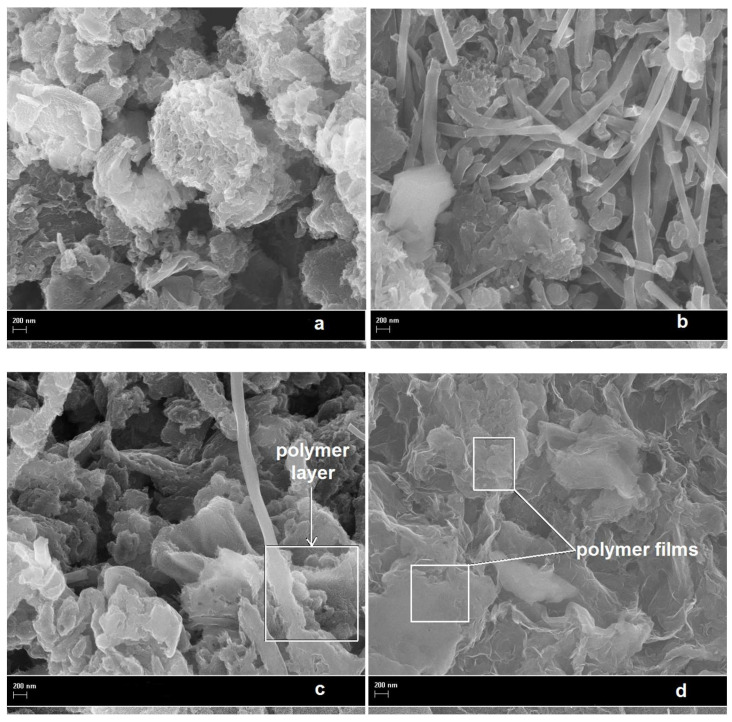

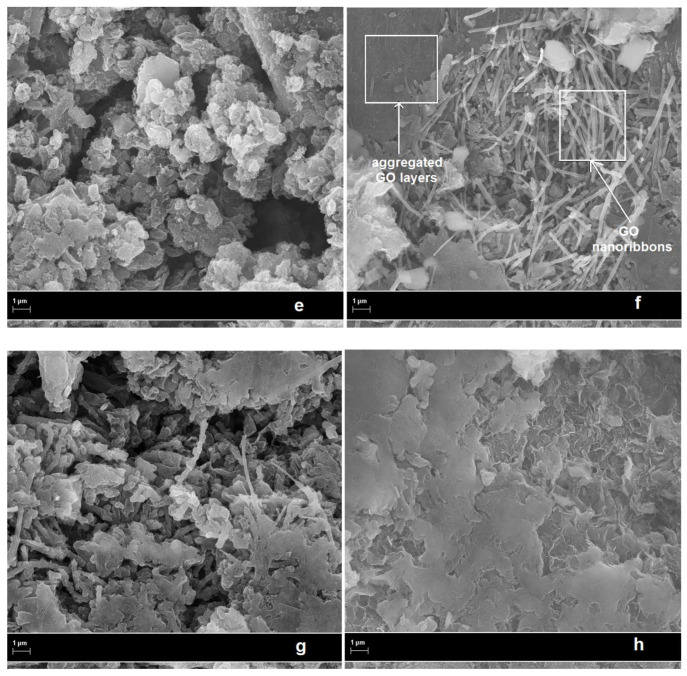
SEM image of PDACB and its composites with 12 wt.% GO: PDACB (**a**,**e**); PDACB/GO-1 (**b**,**f**); PDACB/GO-2.2 (**c**,**g**); PDACB/GO-3.2 (**d**,**h**); (**a**–**d**)—scale size 200 nm, (**e**–**f**)—scale size 1 μm.

Formation conditions of nanocomposites based on PDACB and GO determine not only their composition and structure but also their morphology. Figure 7 shows SEM micrographs of PDACB and PDACB-based hybrid nanocomposites. PDACB (Figure 7a,e) was characterized by a cluster relief morphology formed by irregular aggregates and individual nanoparticles of indefinite shape [78,79].

Figure 7b,f show the presence of GO nanoribbons in the composition of the PDACB/GO-1 nanocomposite. The surface structure of PDACB/GO-1 is represented by alternating areas of accumulation of GO nanoribbons fragments of various lengths and by aggregated GO layers with polymer agglomerates of various sizes [62,80,81].

When PDACB/GO-2.1 and PDACB/GO-2.2 nanocomposites were synthesized via in situ oxidative polymerization of DACB in the presence of GO, a polymer layer of fine texture was formed on the surface of the template, as shown in Figure 7c,g.

Hybrid PDACB/GO-3.1 and PDACB/GO-3.2 nanocomposites were synthesized under prolonged heating at 85 °C until the complete removal of the solvent. As a result, nanocomposites were formed as thin films of non-specific morphology (Figure 7d,h).

### 3.2. Electrical Conductivity of Composites Based on PDACB and OG

The dependence of alternating current (ac) conductivity σ_ac_ on frequency is described by the following equation:

σ_ac_ = σ_dc_ + Aω^n^,

where ω = 2πf is angular frequency (f—denotes frequency), σ_dc_—part of electrical conductivity of the sample at constant external current, equal to σ_ac_ (with ω tending to 0), n—power law exponent, and A—temperature-dependent coefficient [82,83,84]. 

**Figure 8:**
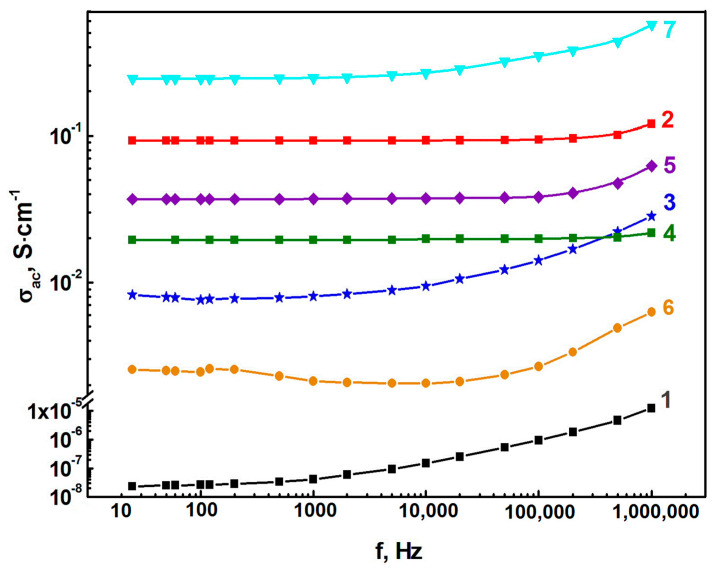
Frequency dependences of electrical conductivity: GO (1), PDACB (2), PDACB/GO-1 (3), PDACB/GO-2.1 (4), PDACB/GO-2.2 (5), PDACB/GO-3.1 (6), PDACB/GO-3.2 (7).

Figure 8 shows the dependence of the electrical conductivity of PDACB and PDACB-based composites synthesized via the methods described above on frequency in the frequency range of 25–10^6^ Hz.

Table 1 provides the values of σ_ac_, σ_dc_, n and A calculated by approximating experimental data. As can be seen from Figure 8, the form of curve 1, which characterizes the frequency dependence of PDACB conductivity, is typical of conductive polymers. The value n = 0.74 and was located in the range 0 < n < 1, which corresponds to the hopping mechanism of polymer conductivity [82,83,84]. Judging by the values of parameter n for PDACB/GO composites, the conduction mechanism did not change, regardless of the formation method.

Low conductivity values of the PDACB/GO-1 composite obtained by mixing the prepared polymer and GO were caused both by the presence of a non-conductive component, namely GO (Figure 3), and a decrease in the degree of polymer doping and an increase in the number of defective structures due to ultrasonic treatment.

Although, as mentioned above, GO participated in the process of in situ oxidative polymerization during the preparation of PDACB/GO-2.1 and PDACB/GO-2.2 nanocomposites, its complete reduction to the conductive RGO state did not occur. This was indicated by the low conductivity values of PDACB/GO-2.1 and PDACB/GO-2.2 nanocomposites, as well as the absence of reflection peaks of GO and RGO (Figure 3, diffractograms 4 and 5).

Under thermal treatment of the suspension of previously obtained PDACB and GO in DMF, the polymer was dedoped, and GO was reduced. The polymer then took its basic non-conductive form. Therefore, the conductivity of PDACB/GO-3.1 can only be explained by the presence of reduced GO, whose content in the non-conductive polymer matrix was too low. In the case of PDACB/GO-3.2, the increase in conductivity was influenced by the following reasons: on the one hand, the dedoping effect was less pronounced due to a decrease in the amount of polymer in the composite, and on the other hand, the effect of RGO increased, which was indicated by a reflection peak in the region of 2θ~42° (Figure 3, diffractogram 7). However, the reduction of GO was not complete. This was indicated by a low-intensity, vague reflection peak of GO shifted to the region of higher 2θ. Unreduced GO causes secondary doping of PDACB of a more elongated structure due to the action of the solvent, thereby providing an additional contribution to the conductivity [27,85].

### 3.3. Thermal Stability of Nanocomposites Based on PDACB and GO

**Figure 9:**
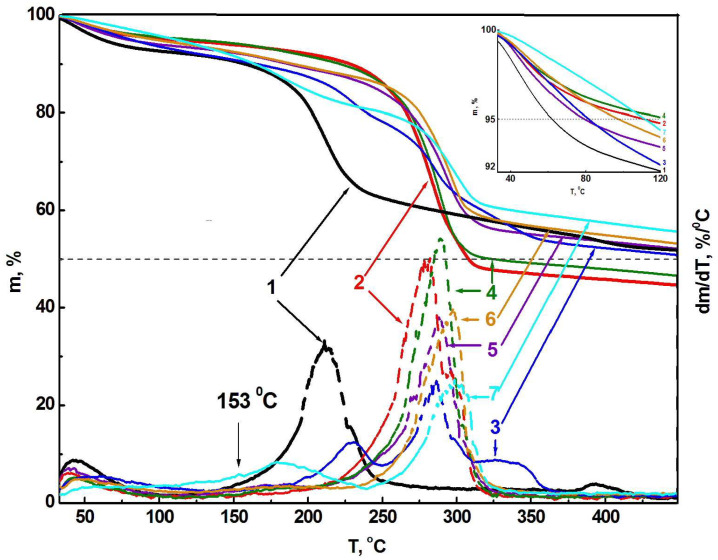
TGA–DTG curves of PDACB, GO and PDACB/GO composites: PDACB (1); GO (2); PDACB/GO-1 (3); PDACB/GO-2.1 (4); PDACB/GO-2.2 (5); PDACB/GO-3.1 (6); PDACB/GO-3.2 (7).

Figure 9 shows temperature dependence of weight change (TGA) and differential thermogravimetric (DTG) curves of the PDACB polymer and PDACB/GO nanocomposites obtained by various methods. The results of thermogravimetric analysis are presented in Table 2.

PDACB, PDACB/GO-2.1 and PDACB/GO-2.2 were characterized by a two-step thermal degradation process in the temperature range of 30–450 °C. Weight loss in the low temperature range (30–100 °C) was due to the removal of moisture and dopant (Cl^−^), similar to what is observed in the case of PANI/GO composites [3,77]. According to DTG, the second step occurred at the temperature range of 220–320 °C and was associated with the removal of low-molecular-weight polymerization products of DACB, residues of PDACB dopant and oxygen-containing groups of GO in composites [3,86,87,88,89,90,91,92,93]. The maximum process rate was achieved at 286.2 °C for PDACB, 288.5 °C for nanocomposites and 293.7 °C for PDACB/GO-2.1 and PDACB/GO-2.2, respectively. The increase in T_max.DTG_ and decrease in the rate of mass loss in PDACB/GO-2.2 were due to the higher content of GO functional groups in the sample, which enhanced the interaction between the polymer and the GO, as evidenced by the results of spectral studies. The region of 320–450 °C characterized the beginning of the polymer chain degradation [77,87,88,89].

A more complex picture was observed in the case of the PDACB/GO-1 nanocomposite. As can be seen in the DTG curve (Figure 9, curve 3), weight loss occurred in three stages. A new peak appeared at T_max.DTG_ = 227.3 °C, corresponding to the removal of oxygen-containing groups of GO. Its shift to the region of higher temperatures than in the case of GO (T_max.DTG_ = 210.8 °C) (Figure 9, curve 1) indicates a strong interaction between GO and PDACB, confirming the formation of a hybrid composite material [4]. The absence of a peak in this temperature range in the case of PDACB/GO-2.1 and PDACB/GO-2.2 nanocomposites is consistent with the formation of a polymer on the surface of GO layers. At the stage corresponding to the removal of low-molecular-weight PDACB products, the maximum decomposition rate was observed at T_max.DTG_ = 286.3 °C.

A low-intensity shoulder (310–355 °C) with a constrained maximum at ~330 °C indicates a slight increase in the thermal stability of PDACB/GO-1. This is associated with the presence of a certain number of cross-linked fragments in the PDACB structure [59] that were formed during the production of composites by mixing PDACB and GO, which was confirmed by spectral analysis data. However, generally, when heated to 450 °C, the weight loss in the PDACB/GO-1 sample (49.15%) increased compared to the weight loss in the PDACB/GO-2.2 sample (47.89%) with the same amount of GO.

For PDACB/GO-3.1 and PDACB/GO-3.2 composites, the maximum removal rate of low-molecular-weight DACB oligomers was achieved at T_max.DTG_ = 297.22 °C and 298.4 °C, respectively. The decrease in intensity of peaks in the low-temperature region was due to the absence of a dopant in the dedoped form of the polymer in PDACB/GO-3.1 and PDACB/GO-3.2 composites (Figure 9, curves 6 and 7). The weight loss observed in the temperature range of 130–230 °C with the maximum weight loss rate at T_max.DTG_ ~179 °C may be explained by the removal of both the remaining solvent (DMF, T_boil_ = 153 °C) from the surface of the composite, which was evidenced by a small peak at T_max.DTG_ = 153 °C, and the solvent which penetrated the interplanar space of GO together with PDACB oligomers dissolved in it [94,95,96]. Additionally, the removal of labile oxygen-containing epoxy, hydroxyl and carboxyl groups [90,91,92] located on basal planes and edge surfaces of GO [70,93] can occur.

Thus, in composite materials based on PDACB and GO obtained by the described methods, an increase in thermal stability was observed, which was demonstrated by the data of Table 2. Higher characteristics of thermal stability showed samples with mixed composition—GO and RGO—where functional oxygen-containing groups of GO strengthened the interaction between the components of the composite. 

## 4. Conclusions

Hybrid nanocomposites based on PDACB in salt form and GO were obtained for the first time. It was established that conditions for the preparation of hybrid nanocomposites play a significant role in the formation of their composition and chemical structure and determine their functional properties. 

It was found that composites obtained by mixing PDACB and GO were characterized by strong interactions between PDACB and GO, both due to electrostatic interaction of oppositely charged fragments and due to the formation of hydrogen bonds and π-stacking.

In the synthesis of PDACB/GO composites via in situ oxidative polymerization of DACB fixed on GO, the latter participated in the redox process, which initiated the polymerization of DACB with a simultaneous reduction of GO, and a polymer layer was formed on the surface of GO nanoribons. When nanocomposites were obtained by heating a suspension of preformed PDACB and GO in DMF with solvent removal, a mixture of PDACB/GO and PDAHCB/RGO nanocomposites was formed, where PDACB was present in the basic non-conductive form. 

The electrical conductivity of nanocomposites depends on the preparation conditions and were in the range of 2 × 10^−3^ to 2.3 × 10^−1^ S⋅cm^−1^. Thermal degradation of nanocomposites in the range of 30–450 °C included the processes of dedoping as well as the removal of moisture, low-molecular-weight polymerization products and oxygen-containing groups of GO. Regardless of the preparation method, all nanocomposites showed an increase in thermal stability compared to PDACB. 

The hybrid nanomaterials studied by us appear promising as electrode materials for energy storage devices.

## Figures and Tables

**Table 1:** Electrical conductivity of PDACB/GO nanocomposites.

Sample	σ_dc_, S⋅cm^−1^	σ_ac_ (25 Hz), S⋅cm^−1^	σ_ac_ (1 MHz), S⋅cm^−1^	n	A, (rad⋅c^−1^)^−n^
PDACB	9.1 × 10^^−2^^	9.3 × 10^^−2^^	1.2 × 10^−1^	0.74	2.6 × 10^−7^
PDACB/GO-1	7.6 × 10^−3^	7.9 × 10^−3^	2.8 × 10^−2^	0.52	7.4 × 10^−6^
PDACB/GO-2.1	1.9 × 10^−2^	1.95 × 10^−2^	2.2 × 10^−2^	0.71	3.8 × 10^−8^
PDACB/GO-2.2	3.8 × 10^−2^	3.8 × 10^−2^	6.2 × 10^−2^	0.96	7.9 × 10^−9^
PDACB/GO-3.1	2.0 × 10^−3^	2.1 × 10^−3^	6.3 × 10^−3^	0.75	3.6 × 10^−8^
PDACB/GO-3.2	2.3 × 10^−1^	2.4 × 10^−1^	5.6 × 10^−1^	0.51	1.1 × 10^−4^

**Table 2:** Thermal properties.

Sample	T_5%_, °C	T_50%_, °C	T_max.DTG_, °C	m_res._, %
PDACB	110.7	307.9	286.2	44.7
GO	61.9		210.8	51.8
PDACB/GO-1	81.4	>450	227.3	50.8
			286.3	
			~330	
PDACB/GO-2.1	123.1	324.3	288.5	46.6
PDACB/GO-2.2	79.1	>450	293.2	52.1
PDACB/GO-3.1	97.2	>450	297.7	53.2
PDACB/GO-3.2	111.4	>450	178.8	55.6
			298.4	

m_res_, %—residual weight of the sample at 450 °C; T_max.DTG_—temperature at which the maximum change in weight occurred.

## Data Availability

The data presented in this study are available on request from the corresponding author.
